# Carbon Dots for Cancer Theranostics: Synthesis Strategies, Luminescence Properties, and Advances in Bioimaging‐Guided Diagnosis and Therapy

**DOI:** 10.1002/chem.202503605

**Published:** 2026-02-07

**Authors:** Zekun Yan, Yupeng Liu, Jinyun Tan, Songnan Qu

**Affiliations:** ^1^ Joint Key Laboratory of Ministry of Education Institute of Applied Physics and Materials Engineering (IAPME) University of Macau Macau SAR China; ^2^ Zhuhai UM Science and Technology Research Institute University of Macau Zhuhai China; ^3^ Huashan Hospital Fudan University Shanghai China; ^4^ Shanghai Key Laboratory of Hydrogen Science & Center of Hydrogen Science School of Materials Science and Engineering Shanghai Jiao Tong University Shanghai China; ^5^ MOE Frontier Science Centre for Precision Oncology University of Macau Macao SAR China

**Keywords:** bioimaging, cancer theranostics, carbon dots, diagnosis, therapy

## Abstract

Carbon dots (CDs) have emerged as a transformative class of luminescent nanomaterials for biomedical applications, offering superior biocompatibility, photostability, and tunability compared to conventional organic dyes and inorganic quantum dots. This review distinguishes itself by establishing a critical “Structure‐Property‐Application” feedback framework. We comprehensively summarize the rational design of CDs, specifically elucidating how core size, surface chemistry, and heteroatom doping can be precisely engineered to tailor optical emission into the near‐infrared II window. Furthermore, the versatile roles of CDs in cancer theranostics are critically discussed, encompassing their utility in subcellular tracking, image‐guided surgery, and multifunctional therapeutic platforms such as photothermal therapy (PTT), photodynamic therapy (PDT), and targeted drug delivery. Finally, we address the current challenges regarding scalability, reproducibility, and biosafety, providing a perspective on the future roadmap for the clinical translation of high‐performance CD‐based agents.

## Graphical abstract

Lighting up the path to precision oncology: This review comprehensively summarizes the rational design of carbon dots (CDs), elucidating how core size, surface chemistry, and heteroatom doping dictate their luminescence mechanisms. Special emphasis is placed on engineering NIR‐II emissive CDs for deep‐tissue imaging. Furthermore, the review highlights the transformative potential of multifunctional CDs in image‐guided surgery and synergistic cancer theranostics, paving the way for their clinical translation.

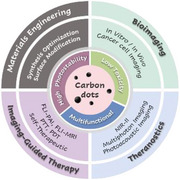

## 1 Introduction

Early and precise tumor diagnosis relies heavily on advanced imaging technologies and high‐performance imaging agents. Over the past decades, numerous inorganic nanomaterials, including semiconductor quantum dots, metal nanoclusters, and rare‐earth‐doped particles, have been extensively explored as contrast agents for tumor imaging and image‐guided therapy. Despite their excellent luminescence and structural stability, issues such as complicated synthesis, limited biodegradability, and potential long‐term toxicity pose significant challenges for further clinical translation. Meanwhile, organic fluorescent dyes suffer from low photostability, rapid photobleaching, and poor signal‐to‐noise ratio in deep‐tissue imaging, which greatly restricts their diagnostic accuracy. Therefore, the development of safe, efficient, and multifunctional imaging materials remains a critical research priority.

Carbon dots (CDs), a burgeoning class of zero‐dimensional carbon nanomaterials with sizes typically below 10 nm, have emerged as a promising solution to these challenges. Since their first discovery in 2004 during the purification of single‐walled carbon nanotubes [1], CDs have attracted intense scientific interest due to their unique structural and physicochemical features. Benefiting from diverse carbon cores and tunable surface chemistry, CDs can be generally categorized into graphene quantum dots [2], carbon quantum dots [3], carbon nanodots [4], and carbonized polymer dots [5]. Compared with conventional inorganic nanoprobes, CDs exhibit outstanding advantages, including facile and cost‐effective synthesis, excellent aqueous dispersibility, low toxicity, and easy surface functionalization [6, 7, 8, 9]. Moreover, their remarkable optical characteristics, such as strong and tunable fluorescence, high photostability, excitation‐dependent emission, and resistance to photobleaching [10], enable real‐time visualization of biological processes with minimal disruption.

**FIGURE 1:**
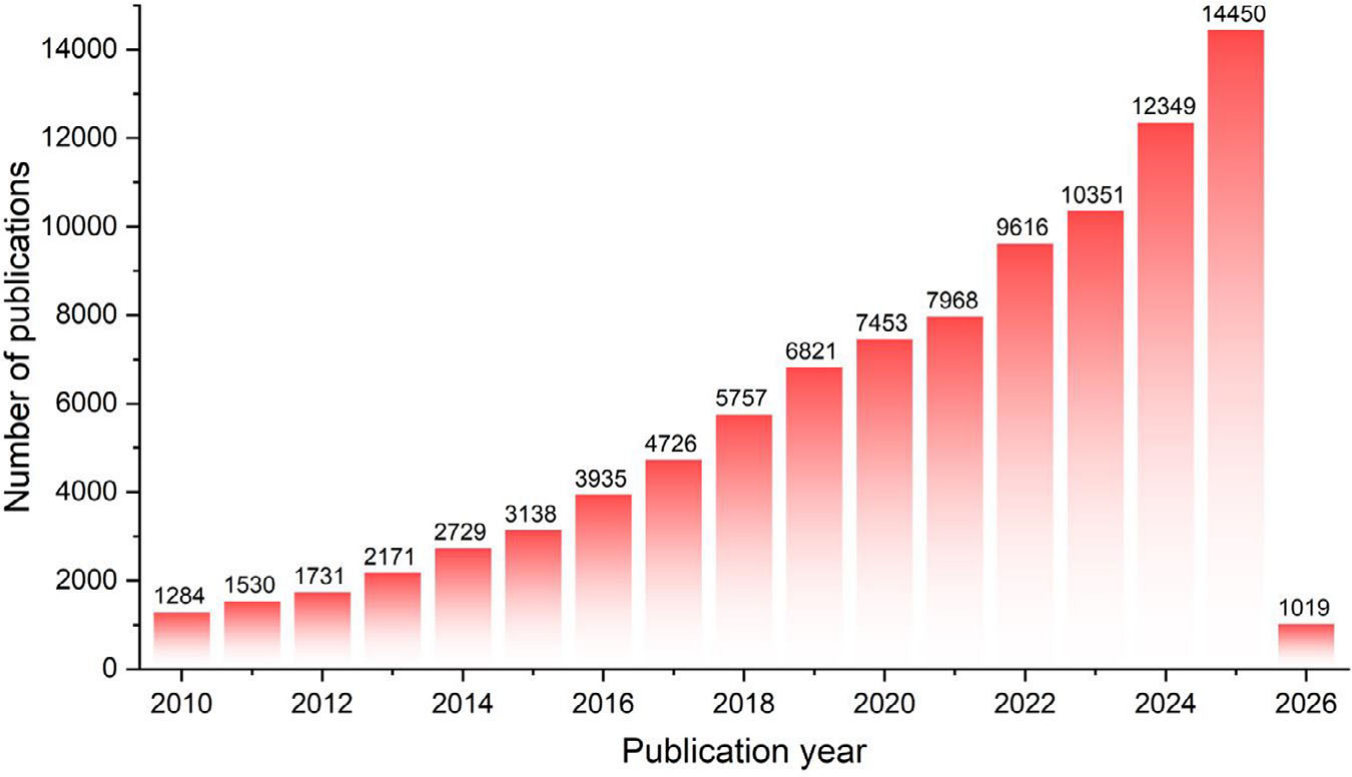
Annual number of publications related to carbon dots for cancer bioimaging and theranostics from 2010 to 2026. (Data *source*: Web of Science Core Collection, keywords: “carbon dot” OR “carbonized polymer dot” OR “carbon nanodot” OR “graphene quantum dot” OR “carbon quantum dot” AND “tumor diagnosis”).

Importantly, continuous progress in structural engineering and surface modification has successfully extended the fluorescence of CDs into the near‐infrared (NIR) window, particularly the NIR second window (NIR‐II, 1000–1700 nm) [11, 12], where biological tissues exhibit lower scattering and absorption. CDs operating in this window can achieve deeper tissue penetration, improved spatial resolution, and superior imaging contrast, making them highly suitable for tumor visualization and guided therapeutic interventions [13]. Furthermore, by combining CDs with other functional materials, magnetic nanoparticles, or radionuclides, multimodal imaging capabilities have been achieved, enabling complementary imaging techniques such as magnetic resonance imaging (MRI), positron emission tomography (PET), and fluorescence imaging [14, 15, 16, 17]. These advancements highlight the growing potential of CDs as multifunctional, high‐performance imaging agents in modern biomedical and therapeutic applications. To quantify the rapid evolution of this field, we conducted a statistical analysis of publications indexed in the Web of Science Core Collection from 2010 to 2026. As illustrated in Figure 1, research output focusing on the topic of “carbon dots in cancer theranostics” has exhibited an exponential growth trajectory and explosive growth in recent years. This trend underscores the urgent need for a comprehensive review to consolidate recent breakthroughs and guide future clinical translation.

**SCHEME 1:**
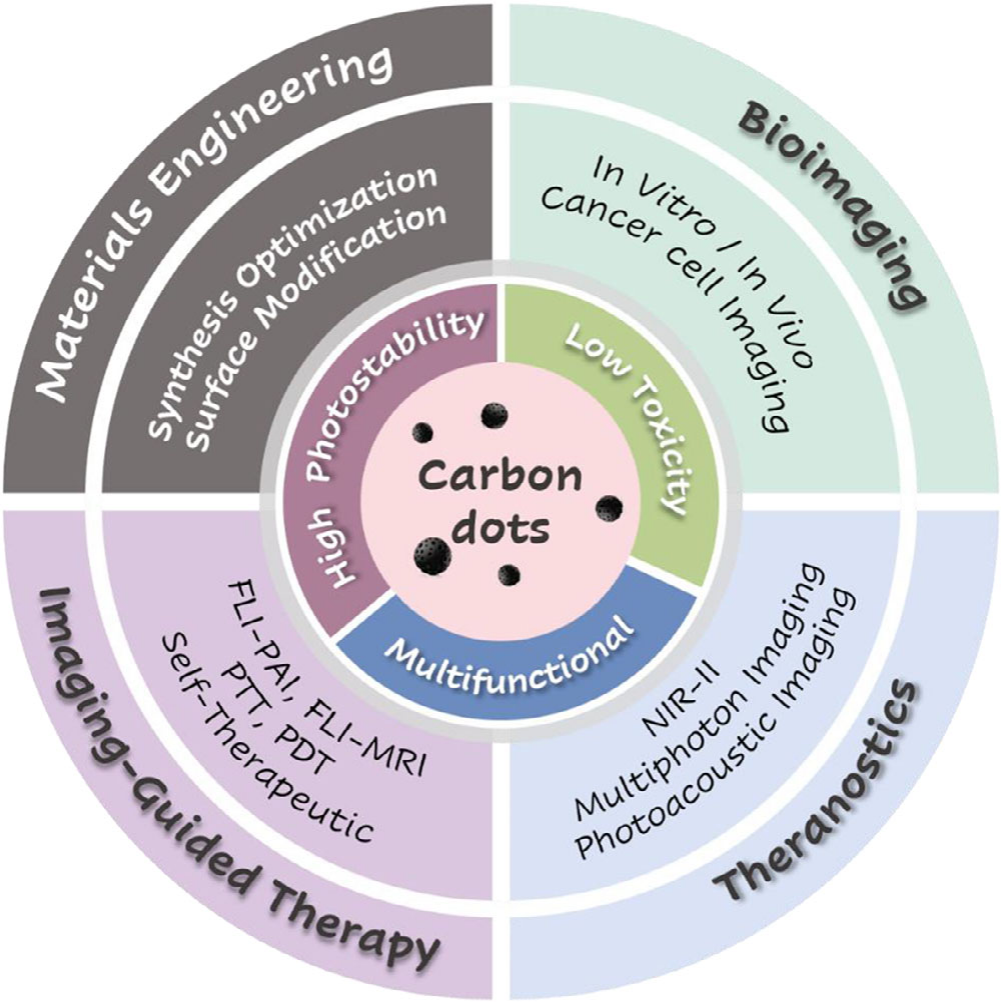
Schematic diagram of the biologically inspired material structure optimization and the multifunctional properties and broad applications of carbon dots across bioimaging, theranostics, and imaging‐guided therapy.

In this review, we summarize recent advances in the synthesis strategies of CDs and discuss how structural and surface engineering tailor their optical behavior. We further highlight representative applications of CDs in tumor bioimaging and imaging‐guided therapy, followed by insights into current challenges and future perspectives (Scheme 1). Unlike previous reviews that broadly categorize CDs applications, this review aims to provide a focused analysis of the bottleneck of deep‐tissue imaging. We critically examine the transition from bench‐top synthesis to NIR‐II clinical potential, aiming to provide guidance for the rational design of high‐performance CDs‐based theranostic agents with improved clinical translation potential.

## 2 Synthesis, Structure, Surface Chemistry, and Luminescence Properties

### 2.1 Synthetic Approaches and Structural Control

**FIGURE 2:**
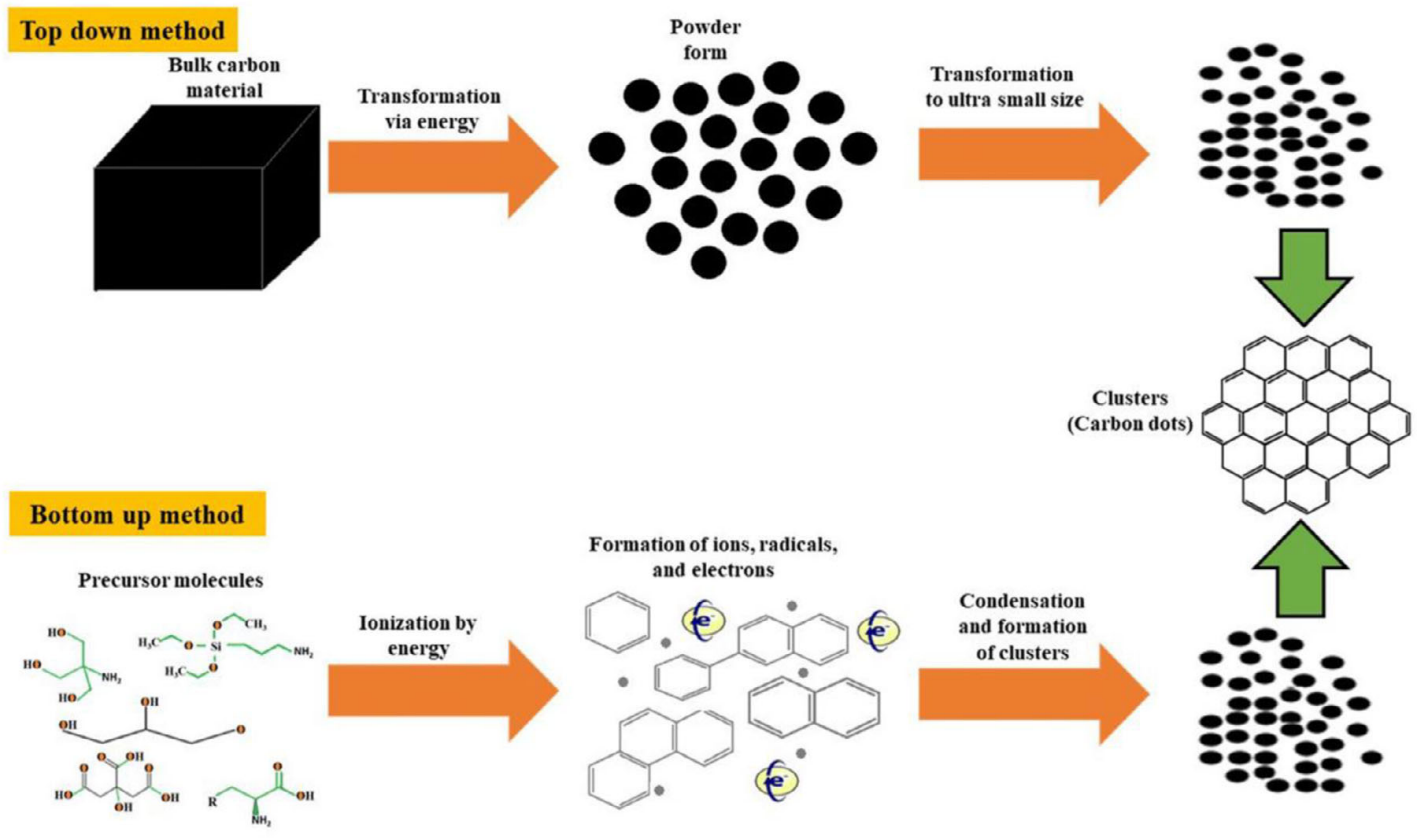
Scheme showing the top‐down and bottom‐up synthesis method of CDs. Adapted with permission from ref [27]. Copyright 2019, Springer Nature.

The synthesis of CDs can be categorized into two approaches: top‐down and bottom‐up (Figure 2). Each of these approaches offers distinct pathways for structural control [6]. Top‐down methods, such as arc discharge, laser ablation, and electrochemical or chemical oxidation, involve fragmenting bulk carbon materials into nanoscale particles [18, 19, 20, 21]. These techniques usually result in the production of CDs with crystalline domains and a relatively uniform size distribution [22] but often require harsh reaction conditions and specialized equipment.

By contrast, bottom‐up strategies involve the construction of CDs from molecular precursors through processes such as hydrothermal or solvothermal treatment, microwave‐assisted carbonization, pyrolysis, or template‐assisted synthesis [23]. These methods facilitate enhanced control over parameters such as particle size, surface functionalization, and heteroatom doping. For instance, the process of hydrothermal synthesis, utilizing biomass‐derived precursors, has been demonstrated to yield oxygen‐ or nitrogen‐rich CDs, which are characterized by a substantial number of functional groups [24]. Microwave‐assisted routes offer rapid reaction kinetics [25], although they may compromise uniformity in some cases [26].

### 2.2 Luminescence Mechanisms

The photoluminescence (PL) origin of CDs is intricate and remains a subject of ongoing debate; however, it is generally universally categorized into four primary mechanisms: core‐state transitions, surface‐state emission, molecular‐state fluorescence, and crosslink‐enhanced emission (CEE). These mechanisms often function synergistically rather than in isolation, defining the optical behavior of the resulting nanomaterials.

**FIGURE 3:**
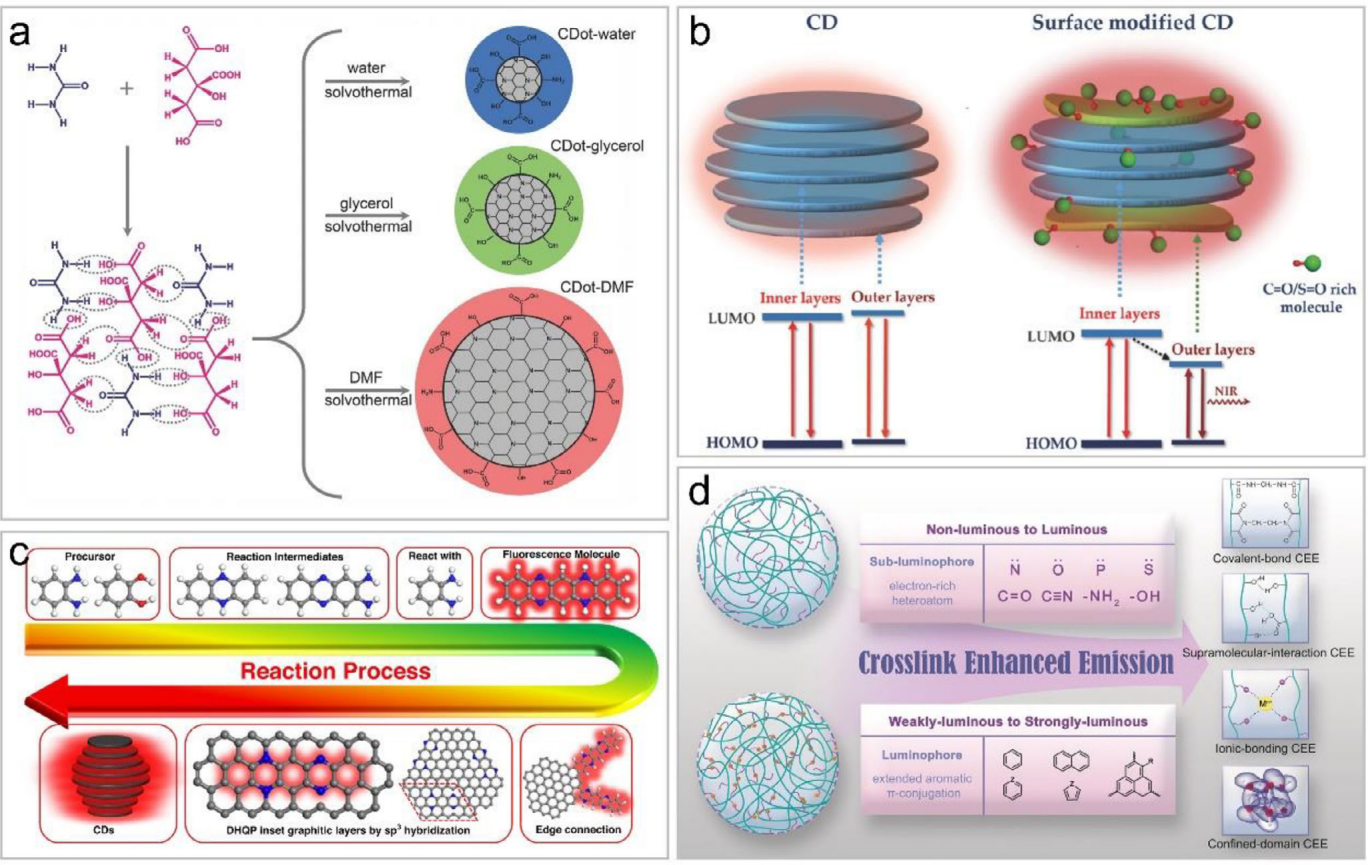
Illustration of the primary luminescence mechanisms of CDs. (a) Size‐dependent fluorescence evolution of CDs synthesized from citric acid and urea, where modulating the solvent environment leads to particle growth and a concomitant bathochromic shift in emission. Reproduced with permission from Ref. [28]. Copyright 2017, Wiley‐VCH. (b) Comparative energy band diagrams and structural models of pristine CDs (left) versus surface‐engineered‐CDs functionalized with electron‐withdrawing C═O/S═O groups (right), highlighting the introduction of new surface energy levels (indicated by red/green spheres). Reproduced with permission from Ref. [30]. Copyright 2018, Wiley‐VCH. (c) Proposed mechanism for molecular‐state fluorescence, originating from fluorophores formed in situ during the reaction between o‐phenylenediamine and o‐catechol. Reproduced with permission from Ref. [34]. Copyright 2022, Springer Nature. (d) Schematic representation of the Crosslink‐Enhanced Emission (CEE) strategy, which amplifies luminescence by restricting the intramolecular motion of potential luminophores within the polymer matrix. Reproduced with permission from Ref. [36]. Copyright 2020, Wiley‐VCH.

Core‐state luminescence is derived from the quantum confinement effect (QCE) within the conjugated *sp^2^* carbon domains (Figure 3a). Analogous to semiconductor quantum dots, the bandgap of CDs is inversely proportional to the size of the conjugated π‐system [28]. Expanding these domains, for instance, by optimizing solvothermal solvents to promote carbonization, effectively narrows the HOMO‐LUMO gap, thereby enabling a redshift in emission from the blue to the red region [29].

Surface‐state luminescence, in contrast, is dominated by the radiative recombination of excitons at surface defect sites as illustrated in Figure 3b [30]. The functionalization of CDs with electron‐withdrawing groups (e.g., C═O, S═O) or varying degrees of oxidation introduces discrete energy levels within the intrinsic bandgap [31, 32]. These surface states act as exciton traps that can facilitate lower‐energy emissions, which is a critical strategy for engineering NIR‐I emissive probes without excessively enlarging the particle size.

Molecular‐state fluorescence attributes the emission to small organic fluorophores (e.g., IPCA, DHQP) that are formed in situ during the pyrolysis of precursors [33]. These fluorophores may be covalently attached to the carbon backbone or physically embedded within the carbon matrix. In many bottom‐up syntheses, the observed high quantum yield and specific spectral features often mirror those of these molecular intermediates as in Figure 3c, suggesting they act as the primary emission centers [34].

Crosslink‐enhanced emission (CEE) provides a rationale for the luminescence of polymer‐based or nonconjugated CDs. As shown in Figure 3d, this mechanism posits that the crosslinking of polymer chains “fixes” the potential luminescence centers (such as amine or amide groups), thereby suppressing the vibration and rotation of molecules [35]. This rigidification minimizes non‐radiative decay pathways, significantly enhancing the PL intensity [36].

Understanding these mechanisms is the prerequisite for the rational design of high‐performance CDs. Through precise regulation of the core size (bandgap engineering), surface defects (trap state engineering), and molecular doping, the emission of CDs can be tailored to span the visible spectrum and successfully extend into the NIR‐I (700–900 nm) and NIR‐II (1000–1700 nm) regions [9]. The utilization of these longer wavelengths is particularly advantageous for deep‐tumor imaging due to the reduced scattering and photon absorption by biological tissues. Furthermore, the versatile structural features allow CDs to be incorporated into multimodal imaging systems, where their fluorescence complements photoacoustic [37] or magnetic resonance modalities [38]. The inherent capacity for photothermal conversion further supports applications in image‐guided therapy, thereby bridging the gap between diagnostic imaging and therapeutic intervention [39, 40].

### 2.3 Structural Features Dictating Luminescence

The photophysical behavior of CDs is governed by a complex interplay between their intrinsic carbogenic core [41] and extrinsic surface states [42], establishing a “core‐shell” electronic structure model. The luminescence mechanisms are primarily dictated by three structural dimensions: the size of the conjugated π‐domains, the degree of surface oxidation, and the specific configurations of heteroatom doping [35, 41].

**FIGURE 4:**
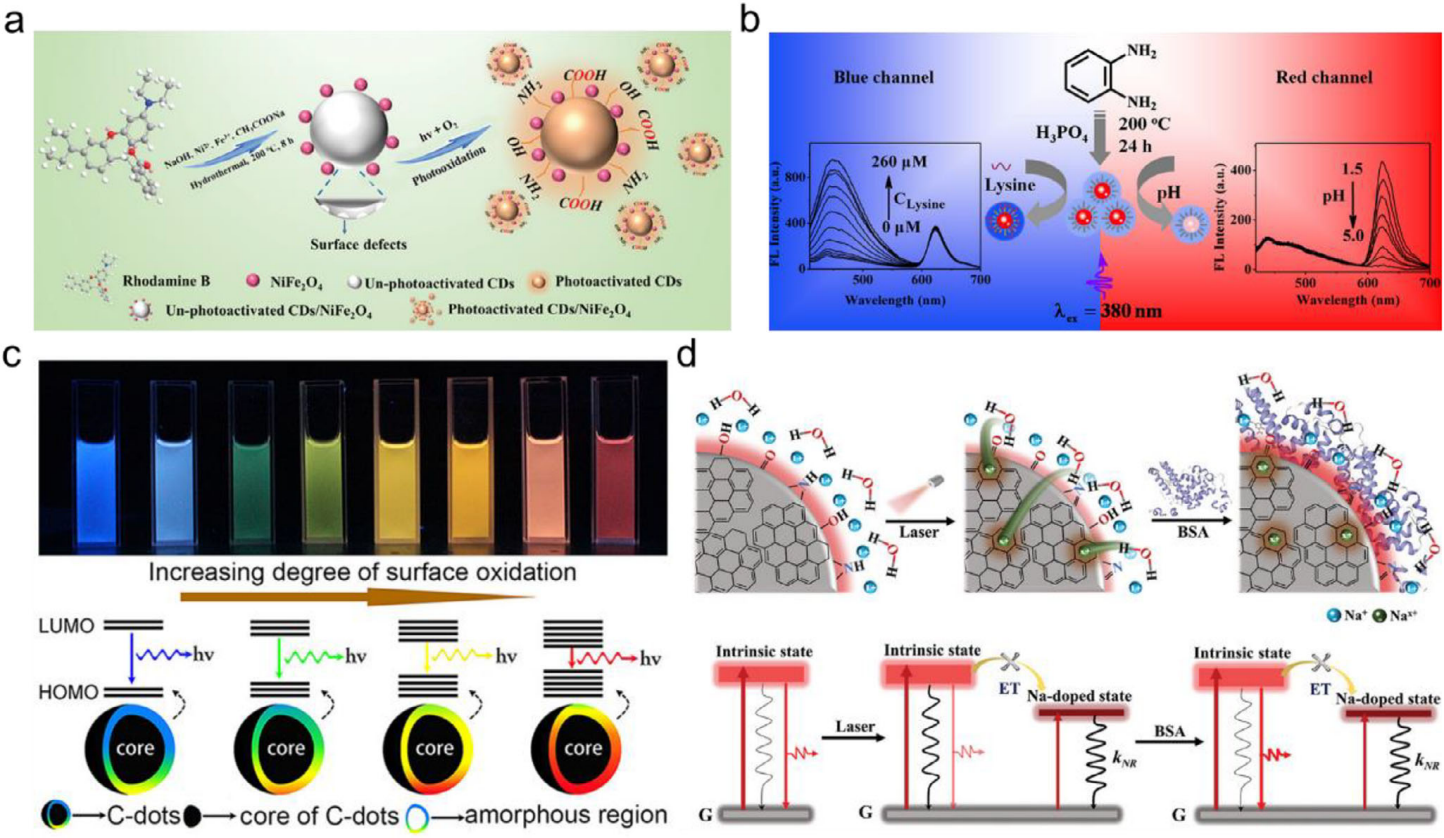
(a) Schematic illustration showing that doping and surface defects alter CDs' luminescence properties. Reproduced with permission [54]. Copyright 2024, Wiley‐VCH GmbH. (b) Schematic illustration of optimizing the structure of CDs to make their luminescence pH sensitive. Reproduced with permission [55]. Copyright 2017, American Chemical Society. (c) Schematic illustration for the tunable photoluminescence of CDs by changing the surface state with different degrees of surface oxidation. Reproduced with permission [49]. Copyright 2015, American Chemical Society. (d) Schematic illustration of the Na doping and bovine serum albumin (BSA) encapsulation into CDs enables NIR‐II luminescence. Reproduced with permission [56]. Copyright 2024 The Authors.

First, the conjugated core size plays a pivotal role through the quantum confinement effect (QCE) [35]. Unlike the overall particle size, it is the extent of the *sp^2^*‐hybridized graphitic domains that determines the intrinsic bandgap (*E*_g_) [43]. According to theoretical calculations and experimental observations, as the conjugated domain size increases, the gap between the Highest Occupied Molecular Orbital (HOMO) and the Lowest Unoccupied Molecular Orbital (LUMO) narrows, resulting in a red shift of the emission wavelength (Figure 4a,b) [44]. This intrinsic state emission, typically arising from π‐π^*^ transitions of the C═C backbone [45], is crucial for achieving stable emission in the near‐infrared (NIR) region, which is highly desirable for deep‐tissue imaging due to minimized photon scattering [46].

Second, surface chemistry exerts a dominant influence, often overshadowing the intrinsic core emission [47]. Surface oxidation and functionalization (e.g., ─COOH, ─OH, ─NH_2_) introduce various energy levels, known as surface defect states, within the bandgap of the CDs [48]. These functional groups create “traps” that capture excitons; the subsequent radiative recombination of these trapped electron‐hole pairs generates fluorescence [8]. Ding et al. [49] demonstrated that the emission color of CDs can be tuned from blue to red solely by controlling the degree of surface oxidation, which introduces lower‐energy surface states closer to the HOMO, thereby reducing the effective transition of energy (Figure 4c). Furthermore, surface moieties are sensitive to the local environment; variations in pH or ionic strength alter the protonation/deprotonation status of these groups, modulating the non‐radiative decay pathways and fluorescence lifetime [50].

Thirdly, heteroatom doping, such as N, S, and P, effectively engineers the electronic structure of CDs (Figure 4d) [51]. Nitrogen doping, the most common strategy, introduces auxiliary energy levels [48]. For instance, graphitic N doping can raise the Fermi level and narrow the bandgap, while pyrrolic or pyridinic N contributes to the formation of edge states, facilitating *n*‐π^*^ transitions [35]. Codoping with sulfur or phosphorus further creates abundant surface defects and alters the electron density distribution, which not only enhances the quantum yield (QY) but also facilitates the redshift of emission toward the NIR‐II window by minimizing the energy gap [52, 53].

### 2.4 Surface Engineering for Functional Enhancement

In addition to intrinsic structural modifications, surface passivation and functionalization have been shown to further enhance the luminescence properties and biological specificity of CDs [48]. The process of passivation with polymers or small molecules has been demonstrated to be an effective method of suppressing non‐radiative recombination [57, 58]. This, in turn, has been shown to result in enhanced brightness and photostability of the material. Covalent conjugation with targeting ligands, including peptides [59], antibodies [60], and glycans [61], facilitates active targeting of cancer cells or pathological tissues.

It is evident that more sophisticated designs incorporate stimuli‐responsive linkers that react to tumor‐specific cues, such as acidic pH [62] or hypoxia [63]. These systems have the capacity to release therapeutic agents while simultaneously providing fluorescence‐based localization, thus demonstrating how surface engineering facilitates the development of integrated theranostic platforms.

In summary, the optical properties of CDs are governed by a complex interplay of size, doping, surface chemistry, and environmental conditions. A comprehensive understanding of these structure‐property relationships is imperative for the design of advanced CDs that are optimized for high‐performance, targeted bioimaging, and theranostics. Crucially, these structural engineering strategies, particularly the surface defect passivation and heteroatom doping discussed above, are closely related to the emission bandgap of CDs and form the basis for their biomedical applications, such as imaging. For example, achieving NIR‐II emission of CDs through doping is a prerequisite for deep tissue imaging.

## 3 CDs in Cancer Bioimaging and Theranostics

### 3.1 In Vitro Cancer Cell Imaging

#### 3.1.1 Tumor Cell Labeling and Tracking

**FIGURE 5:**
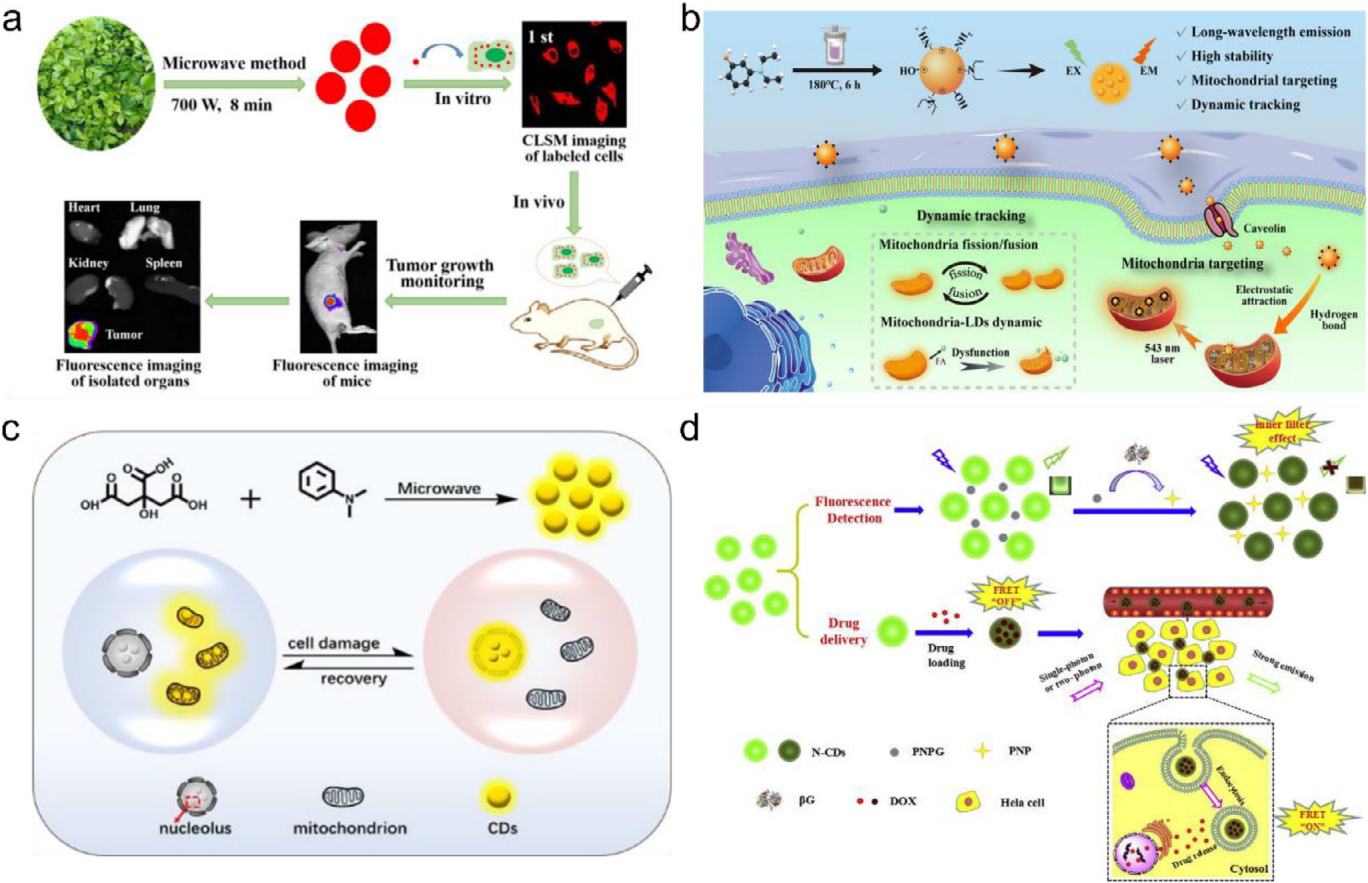
(a) Schematic illustration of the preparation of biomass‐derived carbon dot near infrared fluorescence imaging probes for real‐time cell tracking and tumor growth monitoring. Reproduced with permission [64]. Copyright 2024 The Author(s). Published by the Royal Society of Chemistry. (b) Schematic illustration of the synthetic route of O‐CDs for mitochondrial imaging and long‐term dynamics tracking. Reproduced with permission [68]. Copyright 2023 American Chemical Society. (c) Schematic illustration of the strategy for synthesizing CDs and their shuttling process between mitochondria and the nucleolus in living cells with different statuses. Reproduced with permission [69]. Copyright 2020 American Chemical Society. (d) Schematic illustration of the application of N‐CDs for ultrasensitive detection of β‐glucuronidase based on IFE, visually monitor anticancer drug loading by FRET, and monitor drug delivery by two‐photon fluorescence imaging. Reproduced with permission [70]. Copyright 2018 Elsevier B.V. All rights reserved.

CDs have garnered significant attention as versatile and efficient nanoprobes for the labeling and tracking of tumor cells under in vitro conditions. Owing to their exceptional photostability, high quantum yield, and favorable biocompatibility, CDs facilitate real‐time observation of cellular dynamics with high fidelity. Hu et al. [64] synthesized biomass‐derived red emission carbon dots (RCDs) from the ethanol extract of wintergreen leaves using a one‐step microwave method (Figure 5a). The RCDs exhibited intense red fluorescence in cells even after 13 generations of cell passage, which enabled it to monitor tumor growth in real time for over 18 days. Moreover, the conjugation of CDs with specific targeting ligands significantly improves their selectivity toward particular cancer cell types, thereby enhancing the precision of tumor cell identification and sustained tracking. Li et al. [65] prepared anti‐EpCAM‐NCDs for efficient target recognition using citrate as the carbon source and urea as the passivator and nitrogen source. The EpCAM antibody serves as the target recognition material, capable of specifically binding to EpCAM on the surface of HepG2 cells. After anti‐EpCAM modification, the NCDs surface can specifically recognize EpCAM on tumor cells and enter them. Anti‐EpCAM and NCDs exhibit a complementary relationship. Upon binding to NCDs, anti‐EpCAM gains immunofluorescence. Anti‐EpCAM‐loaded NCDs demonstrate active targeting capability. Despite these advances, surface functionalization often increases synthesis complexity and may alter biostability, indicating the need for standardized and scalable surface engineering strategies.

#### 3.1.2 Subcellular Targeting

The capacity of CDs to localize within specific subcellular organelles has unveiled new prospects for detailed cancer cell imaging. By functionalizing CDs with nuclear localization signals or cationic moieties, they can traverse the nuclear envelope and accumulate selectively within the nucleus. Chen et al. [66] synthesized cationic carbon dots (Y15‐CDs), which have been shown to bind to DNA through electrostatic interactions and a partial insertion binding mode, and to osteosarcoma cells, from 1,2,4,5‐phenanthrene tetramine (Y15) using a hydrothermal method. Cell imaging and cytotoxicity studies have demonstrated that Y15‐CDs exhibit nuclear targeting and anti‐cancer activity. Similarly, modifying with mitochondrial‐affinity groups such as triphenylphosphine (TPP) or adjusting the surface functional groups of CDs by controlling precursors facilitates specific targeting of mitochondria driven by the electrochemical gradient across mitochondrial membranes. Wu et al. [67] constructed a mitochondrial‐targeting nanoprobe (C‐dots‐TPP) by covalently attaching TPP to C‐dots, which had been synthesized from o‐phenylenediamine precursors. This nanoprobe demonstrates sensitivity to peroxynitrite, with its fluorescence quenched via a photoinduced electron transfer (PET) mechanism, and has a detection limit of 13.5nM. This demonstrates excellent mitochondrial targeting and imaging contrast for peroxynitrite in living cells. Xin et al. [68] reported O‐CDs that achieve high colocalization (0.90) with mitochondria (Figure 5b). These O‐CDs enable clear observation of mitochondrial morphology, distribution, and dynamic interactions with lipid droplets during apoptosis and mitophagy in HeLa cells under both physiological and pathological conditions. These studies highlight the utility of CDs in uncovering organelle‐specific mechanisms; however, cytotoxicity induced by strong cationic modifications and potential interference with organelle function remain important considerations for long‐term studies.

#### 3.1.3 CDs in Viability and Toxicity Screening

CDs are increasingly integrated into high‐throughput screening systems for evaluating cancer cell viability and compound‐induced cytotoxicity. Their intrinsic fluorescence permits real‐time, label‐free monitoring of cellular status, including metabolic activity, membrane integrity, and redox homeostasis. Guo et al. [69] synthesized fluorescent carbon dots (CDs) that could shuttle between mitochondria and nucleoli using microwave‐assisted synthesis (Figure 5c). They then used these CDs to visualize cellular vitality in situ. The CDs successfully visualized both hydrogen peroxide (H_2_O_2_)‐induced cellular damage and ascorbic acid‐mediated cellular protection. Gong et al. [70] developed a two‐photon imaging platform based on pyrolytically synthesized nitrogen‐doped carbon dots (N‐CDs) that could be responsive to β‐glucuronidase, enabling simultaneous tumor biomarker sensing and drug uptake evaluation (Figure 5d). Furthermore, incorporating CDs into microfluidic platforms allows for automated, highly sensitive, and rapid cytotoxicity profiling of potential anticancer agents. Wu et al. [71] developed a paper‐based portable disposable cell detection device that utilizes carbon dot nanolabels and aptamers to specifically recognize and capture cancer cells, exhibiting a linear relationship between its electrochemical luminescence signal and cell concentration across a broad range. Furthermore, the sensor can assess the apoptotic effects induced by anticancer drugs by monitoring signal changes, demonstrating potential applications in drug screening. Although these approaches demonstrate strong sensitivity, fluorescence signal drift under prolonged culture and variability in biological environments may hinder reproducibility.

### 3.2 In Vivo Tumor Imaging

#### 3.2.1 NIR‐II Imaging for Deep‐Tissue Penetration

**FIGURE 6:**
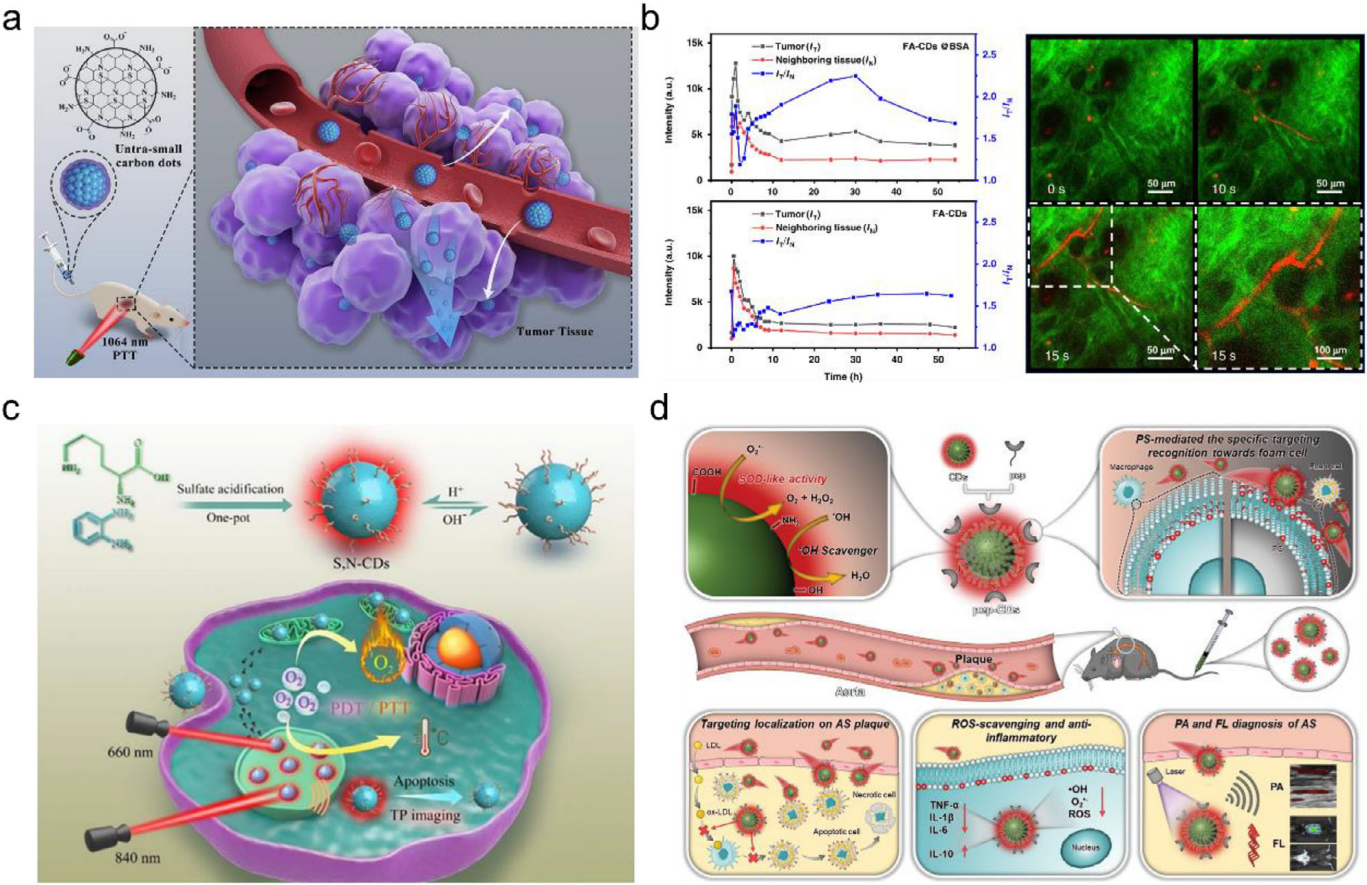
(a) Schematic illustration of ultra‐small carbon dots promoting anticancer efficiency by enhancing tumor penetration in breast cancer under near‐infrared‐II irradiation. Reproduced with permission [72]. Copyright 2022 Elsevier Inc. All rights reserved. (b) Schematic illustration of tumor‐specific accumulation of FA‐CDs@BSA following intravenous injection and two‐photon fluorescence imaging of mouse ear vasculature. Reproduced with permission [73]. Copyright 2022, The Author(s), Springer Nature. (c) Schematic illustration of the S,N‐CDs' synthesis, two‐photon fluorescence imaging of pH, and PDT/PTT synergistic therapy. Reproduced with permission [74]. Copyright 2021 American Chemical Society. (d) Schematic diagram of pep‐CDs with phosphatidylserine targeting function for atherosclerosis theranostics. Reproduced with permission [37]. Copyright 2023 The Authors.

Recent advances in the synthesis of CDs emitting in the second near‐infrared window (NIR‐II, 1000–1700 nm) have substantially improved deep‐tissue imaging capabilities. The reduced scattering and autofluorescence within this spectral region allow for superior spatial resolution and greater penetration depths compared to conventional NIR‐I or visible‐light probes. Yu et al. [72] developed ultra‐small permeable carbon dots (PCDs) that exhibit excellent biocompatibility and photothermal properties within the NIR‐II window (Figure 6a). These PCDs display multicolor fluorescence within the visible spectrum and generate photoacoustic signals upon 808 nm excitation, thus enabling dual‐modality fluorescence/photoacoustic imaging for in vivo localization and distribution analysis. Furthermore, PCDs have been demonstrated to exhibit exceptional capabilities for deep tissue penetration, effectively traversing tumor spheroids and surrounding tissues. CDs with NIR‐II emission thus facilitate clear visualization of deeply seated tumors, improving early detection and longitudinal monitoring of disease progression. However, achieving high quantum yields in the NIR‐II region while retaining favorable clearance profiles remains a key challenge for clinical application.

#### 3.2.2 Two‐Photon and Multiphoton Imaging

CDs exhibiting large two‐photon absorption cross‐sections are ideal candidates for multiphoton imaging. This technique offers several advantages, including reduced photodamage, deeper tissue penetration, and inherent optical sectioning capability for three‐dimensional reconstructions. Zhang et al. [73] synthesized pure red fluorescent carbon dots (FA‐CDs) from citric acid and urea via a one‐step solvothermal method in formic acid (Figure 6b). These were subsequently complexed with bovine serum albumin (BSA) to form FA‐CDs@BSA, exhibiting significantly enhanced multiphoton fluorescence with a PLQY as high as 21.9% in aqueous solution. The application of FA‐CDs@BSA has been demonstrated to be effective in vivo for tumor imaging and two‐photon fluorescence imaging of the ear vasculature in murine models. Bai et al. [74] synthesized S,N‐co‐doped carbon dots (S,N‐CDs) with red‐shifted absorption effects via hydrothermal synthesis using lysine, o‐phenylenediamine, and sulfuric acid as raw materials (Figure 6c). The NIR absorption properties of S,N‐CDs enabled two‐photon emission, which was utilized for two‐photon fluorescence imaging of lysosomal and tumor tissue pH, as well as real‐time monitoring of apoptosis during tumor phototherapy. This technique allows for high‐resolution imaging of tumor morphology and associated vasculature within intact tissue environments, thereby providing critical information for tumor characterization and treatment assessment.

#### 3.2.3 Photoacoustic Imaging of Tumor

Beyond their fluorescent properties, CDs can be engineered as potent contrast agents for photoacoustic imaging (PAI). This technique integrates the use of optical contrast with ultrasound detection, thereby producing high‐resolution images of tumor morphology and microenvironment. Xu et al. [75] synthesized supra‐carbon nanodots (supra‐CNDs) that serve concurrently as contrast agents for PAI and photothermal therapy (PTT). This study demonstrated that intravenous administration of supra‐CNDs enriches at tumor sites via the circulatory system, with tumor accumulation validated through in vivo PAI. Under 655 nm laser irradiation, supra‐CNDs accumulated within tumors effectively mediated PTT, thereby inhibiting tumor growth and prolonging mouse survival. Chen et al. [37]. constructed a targeted theranostic nanoplatform (pep‐CDs) by straightforwardly coupling the phosphatidylserine‐specific peptide CLIKKPF with multifunctional carbon dots (CDs) (Figure 6d). The pep‐CDs exhibited retention of the deep red fluorescence emission and photoacoustic response properties of CDs, thereby enabling dual‐mode PAI and fluorescence imaging‐guided atherosclerosis precision therapy in both in vitro and in vivo settings. Due to their pronounced near‐infrared absorption characteristics, CDs generate substantial photoacoustic signals, thereby enabling precise delineation of tumor boundaries, spatial distribution, and heterogeneous structures. Such detailed characterization is critical for accurate tumor staging, progression tracking, and evaluating responses to various cancer therapies. Nevertheless, the photoacoustic efficiency of CDs still trails behind inorganic alternatives, motivating efforts to enhance absorption cross‐sections and stability under physiological irradiation.

### 3.3 CDs for Cancer Diagnosis and Biosensing

#### 3.3.1 Tumor Microenvironment Sensing

The tumor microenvironment (TME) is characterized by a distinct metabolic profile, primarily driven by aerobic glycolysis, which leads to lactic acid accumulation, extracellular acidosis, and elevated reactive oxygen species (ROS) levels. Unlike traditional organic dyes (e.g., FITC, Rhodamine), which often suffer from fluorescence quenching, photobleaching, or poor stability in hypoxic and acidic conditions, CDs demonstrate superior stability. They can be engineered as “turn‐on” sensors that specifically activate in response to these stress factors, providing a higher signal‐to‐noise ratio in complex physiological environments. CDs have been strategically engineered as responsive probes to monitor these critical biochemical parameters dynamically. During the process of transformation of normal cells into tumor cells, aberrant glycolysis results in a substantial increase in cytoplasmic nicotinamide adenine dinucleotide (NAD^+^, oxidized) levels. Li et al. [76] prepared hydrothermally synthesized nitrogen‐doped carbon dots (N‐CDs) from 2,3‐diaminophenazine as fluorescent probes for imaging aerobic glycolysis. Under physiological conditions, NAD^+^ specifically enhances the photoluminescence intensity emitted by N‐CDs, enabling the identification of a small number (five to ten) of abnormal cells in a spontaneous hepatocellular carcinoma model before tumor tissue develops in situ. The N‐CDs probe has the capacity to differentiate between tumor cells and normal cells. pH‐sensitive CDs show reversible fluorescence changes under acidic TME conditions. Bai et al. [74] synthesized sulfur and nitrogen codoped carbon dots (S,N‐CDs) with red‐shifted absorption effects using lysine, o‐phenylenediamine, and sulfuric acid as raw materials via a hydrothermal synthesis method. Under normal physiological pH conditions, the red fluorescence of S,N‐CDs is barely detectable. Upon entering the tumor microenvironment, the fluorescent signal significantly intensifies, and upon reaching intracellular lysosomes, a distinct fluorescent signal is generated. ROS‐responsive CDs alter their emission upon oxidation. Kim et al. [77] developed an electro‐sensor based on polydiselenide crosslinked polymer dots (PDs). The controlled reaction between the diselenide‐containing PDs and ROS within cells has been shown to trigger the cleavage of diselenide (Se─Se) and the formation of its oxidized form (Se─OH/Se─OOH). This process enhances the fluorescence intensity of PDs, thereby altering the size structure of nanoparticles on the coating surface. Consequently, it enables the selective detection of cancer cells through optical and electrical signals, specifically recognizing the cancer microenvironment. Most studies, however, remain at a proof‐of‐concept stage with limited quantitative correlation to disease staging, calling for improved calibration and in vivo validation. Recent technological advances have further integrated stable nanomaterials with sophisticated devices. For instance, implantable biophotonic devices have been developed for wireless, real‐time monitoring of cancer, utilizing optical signals to modulate treatment dynamically [78]. Furthermore, novel strategies utilizing in situ cross‐linked catechol extracellular barriers have been shown to synergize with therapeutic strategies by blocking tumor mass transport, a concept that complements CD‐based delivery systems [79].

#### 3.3.2 Circulating Tumor Cell Detection

**FIGURE 7:**
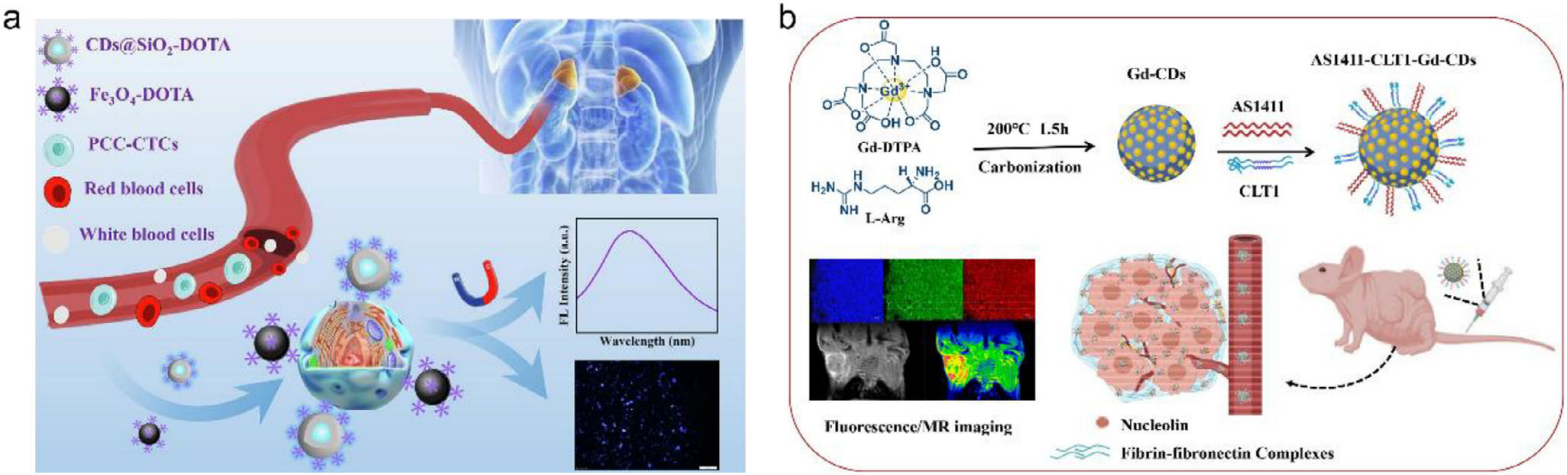
(a) Schematic illustration of capture, separation, and analysis of Pheochromocytoma‐ circulating tumor cells. Reproduced with permission [81]. Copyright 2024 Elsevier B.V. All rights reserved. (b) Schematic illustration of the preparation procedure of AS1411‐CLT1‐Gd‐CDs nanoprobe and its application for dual‐targeted fluorescence/MR imaging of renal cell carcinoma. Reproduced with permission [82]. Copyright 2022 Elsevier B.V. All rights reserved.

Sensitive detection of circulating tumor cells (CTCs) is crucial for early cancer diagnosis and metastatic risk assessment. CDs conjugated with targeting moieties such as antibodies or aptamers enable specific recognition and labeling of CTCs based on surface biomarker expression. The strong and stable fluorescence of CDs permits highly sensitive detection even at low abundances, facilitating quantitative analysis of CTCs in clinical samples for prognostic evaluation. Key challenges include nonspecific binding in complex blood matrices and maintaining probe stability during clinical sample handling. Pheochromocytoma (PCC) is a rare neuroendocrine tumor that is frequently misdiagnosed or overlooked due to its atypical clinical presentation [80]. Octreotide‐2,2',2″,2'″‐ (1,4,7,10 ‐tetraazacyclododecane‐1,4,7,10‐tetrayl) tetraacetic acid (DOTA) is a radionuclide‐labeled somatostatin (SS) analog that has been demonstrated to bind to somatostatin receptors (SSTR) on the surface of PCC cell tumors. Zhao et al. [81] developed a system based on DOTA‐modified magnetic Fe_3_O_4_ and signal‐amplifying CDs@SiO_2_ nanospheres to capture and detect PCC‐CTCs in peripheral blood by binding to the overexpressed somatostatin receptor SSTR2 on PCC cell surfaces (Figure 7a). During the detection process, magnetic capture probes (Fe_3_O_4_‐DOTA) separate and enrich target cells, followed by specific binding of signal probes (CDs@SiO_2_‐DOTA) to form a sandwich structure that outputs fluorescent signals. The designed fluorescent cell sensor demonstrates exceptional sensitivity and selectivity for PCC‐CTCs at concentrations ranging from 5 to 1000 cells mL^−1^, with a detection limit of 2 cells mL^−1^.

#### 3.3.3 Image‐Guided Tumor Margin Delineation

Precise intraoperative identification of tumor margins is essential for complete resection and minimizing relapses. CDs have shown promise as contrast agents for fluorescence‐guided surgery, offering real‐time discrimination between malignant and healthy tissues. Yu et al. [82] developed a dual‐targeting fluorescent/magnetic resonance nanoprobe, AS1411‐CLT1‐Gd‐CDs, by conjugating the peptide CLT1 and the aptamer AS1411 to magnetic fluorescent gadolinium(III)‐doped carbon dots (Gd‐CDs) to precise detection and delineation of renal cell carcinoma (RCC) margins (Figure 7b). AS1411‐CLT1‐Gd‐CDs facilitated targeted imaging of 786‐O RCC cells via three distinct fluorescence channels, achieving quantitative detection of 786‐O cells with a detection limit as low as 552 cells mL^−1^. In addition, the dual‐targeted AS1411‐CLT1‐Gd‐CDs nanoprobe has been shown to precisely delineate tumor margins, is readily excreted via the renal system post‐imaging, and exhibits no significant long‐term toxicity. Their application enables surgeons to visually delineate tumor boundaries, thereby improving resection accuracy and patient outcomes. Future refinement should prioritize real‐time quantification of surgical margins and assessment in large‐animal models.

### 3.4 CDs in Cancer Theranostics

#### 3.4.1 Multimodal Imaging‐Guided Therapy (FLI‐PAI, FLI‐MRI)

**FIGURE 8:**
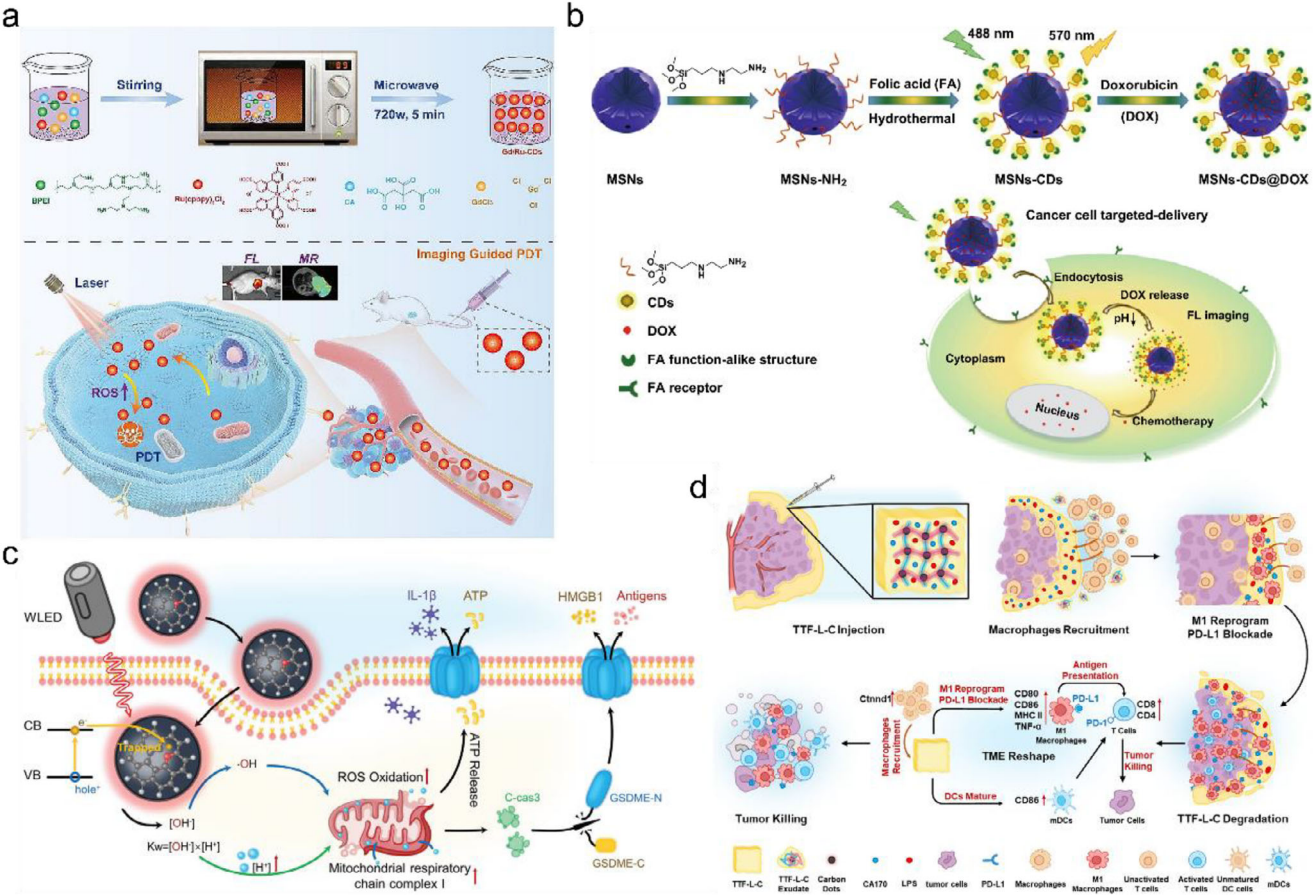
(a) Schematic illustration of microwave‐assisted synthesis of Gd/Ru‐CDs (up) and FL/MR‐guided photodynamic therapy of the tumor (down). Reproduced with permission [83]. Copyright 2024, Springer Nature. (b) Schematic illustration of the preparation procedures and fluorescence imaging‐guided anticancer drug delivery application of the MSNs–CDs nanohybrid. Reproduced with permission [86]. Copyright 2019 The Author(s). (c) Schematic illustration of photocatalytic CDs inducing pyroptosis of cancer cells under WLED irradiation. Reproduced with permission [87]. Copyright 2024 The Author(s). (d) Schematic illustration of the TTF‐L‐C spatiotemporally transformed TAMs to reshape TME for tumor immunotherapy. Reproduced with permission [88]. Copyright 2025 The Author(s).

CDs can be engineered as multifunctional nanoplatforms that combine complementary imaging modalities. For example, doping with magnetic elements confers T_1_ or T_2_ contrast for magnetic resonance imaging (MRI), while retaining fluorescence properties for simultaneous optical imaging. Shi et al. [83] synthesized Gd/Ru bimetallic‐doped fluorescent carbon dots (Gd/Ru‐CDs) via a one‐step microwave‐assisted method using Ru(dcbpy)_3_Cl_2_, citric acid, polyethyleneimine, and GdCl_3_ as precursors. Gd/Ru doping endows the carbon dots with not only excellent MRI performance but also enhanced fluorescence in the red emission region (Figure 8a). Gd/Ru‐CDs exhibit low cytotoxicity, outstanding biocompatibility, and efficient reactive oxygen species (ROS) generation capacity. In vivo experiments demonstrate that Gd/Ru‐CDs enable light‐induced tumor suppression and non‐invasive dual‐mode fluorescence/MRI imaging. Likewise, CDs can serve as dual‐mode agents for both fluorescence and photoacoustic imaging. Ge et al. [84] synthesized red‐emitting carbon dots (C‐dots) from polythiophene benzoic acid, exhibiting broad absorption characteristics in the 400–750 nm wavelength range. Under near‐infrared laser irradiation, the C‐dots exhibit a strong photoacoustic response and high photothermal conversion efficiency (η≈38.5% under 671 nm laser irradiation). C‐dots can simultaneously serve as fluorescent, photoacoustic, and thermotherapy diagnostic agents for cancer diagnosis and treatment in live mice. These integrated systems provide comprehensive anatomical and functional information, improving diagnostic confidence and guiding therapeutic planning.

#### 3.4.2 Imaging‐Guided Therapy: PTT, PDT, and Drug/Gene Delivery With Tracking

CDs exhibit notable potential in imaging‐guided therapeutic applications. In photothermal therapy (PTT), NIR‐absorbing CDs efficiently convert light into heat, inducing hyperthermia‐mediated tumor cell death. Wang et al. [56] synthesized Na‐CDs functionalized with Na^+1^ by introducing sodium carbonate into a citric acid‐urea mixture and performing solvothermal treatment in formic acid. Na doping facilitated the generation of the NIR‐II band in ir‐Na‐CDs. Upon complexation with BSA, ir‐Na‐CDs@BSA exhibited highly efficient red emission in water with a PLQY of 31%. Under 1064 nm laser irradiation (1 W cm^−2^), the photothermal conversion efficiency reached 43%. Furthermore, ir‐Na‐CDs@BSA exhibits extremely low cytotoxicity and demonstrates significant tumor accumulation capacity following intravenous injection. No tumor recurrence was observed in the ir‐Na‐CDs@BSA treatment group after 1064 nm laser therapy.

In photodynamic therapy (PDT), CDs can act as photosensitizers to produce cytotoxic ROS under irradiation. Zhang et al. [85] developed a novel approach to construct donor‐acceptor (D−A) type supra‐carbon dots (supra‐CDs) by introducing 2,3‐dicyanohydroquinone (DCHQ) as an electron‐withdrawing chain segment, followed by post‐solvothermal fusion with red‐emitting carbon dots. The supra‐CDs exhibit a core composed of assembled carbon dots as the D‐type component, while the interface derived from the fused DCHQ serves as the A‐type component. Efficient photoinduced charge separation facilitates the reaction between photoexcited electrons and O_2_, enabling highly efficient generation of O_2_·^−^. This radical then oxidizes key intracellular substrates such as BH_4_ and NADH, demonstrating outstanding photoinduced cell‐killing capability. The increased size of supra‐CDs (approximately 20 nm) enhances their tumor accumulation capacity, enabling satisfactory tumor PDT in a mouse subcutaneous tumor model.

Additionally, the tunable surface chemistry and high loading capacity of CDs make them effective nanocarriers for controlled delivery of chemotherapeutic drugs or genetic material. The intrinsic fluorescence of CDs allows real‐time visualization of agent delivery and treatment response, paving the way for personalized combination therapies. Zhao et al. [86] prepared a nanohybrid material (named MSNs‐CDs) by anchoring folate‐in‐situ‐synthesized carbon dots onto the surface of amino‐modified mesoporous silica nanoparticles (MSNs‐NH_2_) via microwave‐assisted solvothermal reaction (Figure 8b). MSNs‐CDs not only exhibit strong and stable yellow emission but also retain the mesoporous structure, large specific surface area, and excellent biocompatibility of MSNs. Furthermore, they can selectively target cancer cells overexpressing folate receptors. The fluorescent MSNs‐CDs nanohybrid can serve as a fluorescence‐guided nanocarrier for targeted delivery of anticancer drugs (e.g., doxorubicin), thereby enhancing chemotherapy efficacy and reducing side effects.

#### 3.4.3 Self‐Therapeutic CDs and Double Effects

Aside from their cargo‐carrying capacity, some CDs possess inherent therapeutic activities. Emerging evidence suggests that CDs may modulate immune responses, simultaneously inducing tumor cell death and stimulating antitumor immunity. Such double‐effect functionality highlights the expanding role of CDs in multimodal cancer management. Cheng et al. [87] reported photocatalytic CDs effectively induce pyroptosis in cells via the ROS‐mitochondria‐caspase3‐gasdermin E pathway and proton‐driven mitochondrial ATP synthesis pathway upon illumination with white LEDs (Figure 8c). These photocatalytic CDs‐induced pyroptotic cancer cells (PCIP) effectively hyperactivate macrophages (M0 to M1) and upregulate major histocompatibility complex class II expression. In vivo, PCIPs generate immune memory after efficiently delivering antigens to lymph nodes. Upon encountering homologous cancer cells, they rapidly activate specific anti‐cancer immunity, achieving highly effective tumor prevention in melanoma and breast cancer models. Li et al. [88] developed an injectable carbon dots (CDs)‐linked egg white hydrogel termed the tumor‐associated macrophages (TAMs) conversion factory (TTF‐LC) for spatiotemporal manipulation of TAMs (Figure 8d). TTF‐LC achieves spatial enrichment of macrophages through CDs‐induced directed recruitment and upregulation of Ctnnd1 molecular expression. Subsequently, recruited macrophages undergo local reprogramming within TTF‐LC and block the upregulation of PD‐L1. Ultimately, through multistage regulation at spatial, cellular, and molecular levels, TTF‐LC degrades to release immunologically activated M1 macrophages into the tumor site. Additionally, TTF‐LC promotes dendritic cell (DC) maturation, further enhancing T cell activation to remodel the tumor‐suppressive TME. Through peri‐tumor injection, TTF‐LC enhances tumor immunotherapy in both subcutaneous and recurrent 4T1 tumor models with favorable biosafety.

## 4 Summary and Future Perspectives

### 4.1 Summary of Progress

In this review, we have highlighted the transformative evolution of carbon dots (CDs) from fundamental nanomaterials to sophisticated agents for cancer theranostics. We have explored how the rational regulation of synthesis strategies, specifically through heteroatom doping and surface engineering which enables the precise tuning of photoluminescence from the visible to the vital NIR‐II window. Beyond their optical superiority for deep‐tissue imaging, CDs have demonstrated exceptional versatility in sensing the tumor microenvironment, delineating surgical margins, and driving synergistic therapies such as PTT and PDT. These advancements collectively underscore the potential of CDs to surpass traditional organic dyes and inorganic quantum dots, offering a robust platform for next‐generation precision oncology.

### 4.2 Critical Challenges in Clinical Translation

Despite remarkable progress, the translation of CDs from bench to bedside faces distinct bottlenecks compared to clinically approved agents like Indocyanine Green (ICG). Substantial advances in structural engineering, surface passivation, and functionalization have endowed CDs with tunable photoluminescence and versatile biological activity; however, the precise luminescence mechanisms and structure–property relationships are still not fully elucidated, impeding rational molecular‐level design. Unlike established organic dyes, the heterogeneous nature of CDs poses challenges for regulatory approval (FDA/EMA), requiring a rigorous definition of chemical composition and purity profiles.

Another key challenge lies in achieving high tumor‐targeting specificity while minimizing off‐target accumulation and nonspecific uptake. Long‐term biosafety, including toxicity, biodistribution, and clearance behavior, requires systematic evaluation to ensure clinical viability. Specifically, detailed data on long‐term biodistribution and clearance pathways are currently lacking for many novel CDs. Future studies must rigorously differentiate between renal clearance kinetics and hepatobiliary retention, especially for metal‐doped CDs, to prevent potential heavy metal accumulation and chronic toxicity. While laboratory synthesis is facile, achieving industrial‐scale production with batch‐to‐batch consistency remains difficult. Future efforts must focus on standardized protocols compliant with Good Manufacturing Practice (GMP) standards to meet regulatory requirements for clinical trials.

### 4.3 Future Directions and Emerging Trends

Looking forward, the rational design of tumor‐active CDs with well‐defined structures and predictable optical outputs will be pivotal. The development of high‐performance near‐infrared II emissive CDs is expected to enhance deep‐tissue imaging and improve diagnostic precision. Furthermore, multifunctional CD‐based platforms integrating immune modulation, radiosensitization, and combination therapy may offer synergistic therapeutic outcomes. Establishing a clear clinical translation roadmap encompassing preclinical assessment, early‐stage trials, and regulatory harmonization will accelerate their path toward clinical practice. Beyond oncology, the expansion of CD‐based technologies into intraoperative surgical guidance and metastasis detection holds promise for broadening their biomedical impact and establishing CDs as a new class of versatile nanotheranostic agents.

The rapid development of CDs‐based tumor therapeutics suggests emerging directions including CDs‐based immunotherapy, image analysis combined with artificial intelligence (AI), and reactive drug delivery systems tailored to tumor heterogeneity. First, combining CDs‐based imaging technology with AI and deep learning algorithms holds promise for automated delineation of tumor‐normal tissue boundaries and quantitative analysis of molecular imaging data, potentially reducing misdiagnosis by clinicians and minimizing harm to patients. Beyond direct cytotoxicity, CDs represent a frontier in enhancing checkpoint blockade therapy by demonstrating their potential to reshape the immune microenvironment (e.g., macrophage polarization from the M2 to the M1 phenotype). Customized drug delivery systems for heterogeneous tumors can utilize surface functionalization, heterogeneous atom doping, and smart polymer encapsulation, combined with tumor microenvironment (TME) characteristics (such as pH, GSH, enzymes, and redox reactions), to design multiple response mechanisms, achieving precise controlled release and targeted drug delivery. Specifically, this can be approached from the following aspects:

1. AI‐supported CDs imaging can achieve automated tumor identification, grading, and efficacy prediction, supporting the development of individualized treatment plans.
2. By combining photoacoustic, MRI, and CT multimodal imaging, AI enables real‐time data fusion and surgical navigation, improving precision medicine.
3. Constructing a large‐scale CDs imaging database enhances the training accuracy and generalization ability of AI models, promoting clinical translation and multicenter applications.
4. The CDs platform can synergize with immune checkpoint inhibitors such as PD‐1/PD‐L1 and CTLA‐4 to enhance immunotherapy response and overcome the bottleneck of drug resistance in “cold tumors.”
5. As a novel immune adjuvant and vaccine delivery platform, CDs improve vaccine efficacy and reduce toxic side effects, applicable to various scenarios including oncology and infectious diseases.
6. For different types of tumors, by detecting tumor microenvironment‐specific biomarkers such as ROS and NO, customized response mechanisms can be developed to achieve personalized drug release and efficacy monitoring.
7. Combining patient‐specific antigens, tumor microenvironment, and biomarkers, customized CDs‐immune complexes can achieve precision immunotherapy.
8. Based on the characteristics of the patient's tumor microenvironment (pH, GSH, enzymes, etc.), customized multiresponse CDs platforms can achieve personalized precision treatment.

With continuous breakthroughs in synthesis technology, surface engineering, AI algorithms, and multimodal imaging, carbon dot‐based integrated diagnostic, and therapeutic platforms are expected to leap from the laboratory to the clinical setting within the next 10–20 years, becoming a crucial supporting technology for precision medicine and personalized treatment. Their multifunctional integration in areas such as tumor immunotherapy, intelligent imaging navigation, and personalized drug delivery will bring revolutionary changes to the diagnosis and treatment of complex diseases.

## Conflicts of Interest

The authors declare no conflicts of interest.

## Data Availability

The data that support the findings of this study are available from the corresponding author upon reasonable request.
